# Inflammatory cell death: how macrophages sense neighbouring cell infection and damage

**DOI:** 10.1042/BST20220807

**Published:** 2023-01-25

**Authors:** Xiaohui Wang, Larisa I. Labzin

**Affiliations:** Institute for Molecular Bioscience (IMB), The University of Queensland, Brisbane, QLD, Australia

**Keywords:** apoptosis, inflammation, influenza, macrophage, necroptosis, pyroptosis

## Abstract

Programmed cell death is a critical host defence strategy during viral infection. Neighbouring cells deal with this death in distinct ways depending on how the infected cell dies. While apoptosis is considered immunologically silent, the lytic pathways of necroptosis and pyroptosis trigger inflammatory responses by releasing inflammatory host molecules. All these pathways have been implicated in influenza A virus infection. Here, we review how cells sense neighbouring infection and death and how sensing shapes ensuing inflammatory responses.

## Introduction

Programmed cell death (PCD) is an essential physiological process that eliminates non-functional, aged, damaged, or infected cells [1]. Efficient clearance of these dead cells is critical for maintaining tissue and organismal homeostasis [1]. As such, PCD is a crucial pillar of host defence during viral infection. Firstly, PCD removes the viral replication niche and prevents further viral dissemination [2]. Secondly, PCD potentiates and amplifies innate and adaptive immune responses. Over the past few decades, 12 distinct cell death modalities have been identified [3]. These cell death pathways are primarily classified as lytic and therefore ‘inflammatory’; (e.g. pyroptosis, necroptosis) or non-lytic and therefore ‘silent’ (e.g. apoptosis) [4]. Innate immune cells, such as macrophages, clear dying cells and survey tissues for signs of infection and damage. Macrophages sense cellular debris from dying cells as a sign of danger and trigger inflammatory responses. However, apart from triggering or preventing an immune response, little is known about how macrophages sense distinct PCD modalities during infection and respond accordingly beyond the simple lytic *vs* apoptotic paradigm. Here, we examine infection with the common and persistent human pathogen influenza A virus (IAV) as a model to understand how different cell death modalities shape ensuing macrophage inflammatory responses.

IAV is a highly contagious respiratory pathogen that can cause the symptomatic disease known as ‘influenza’ or the ‘flu’. IAV infections cause significant morbidity and mortality, with estimates of up to 36 million illnesses and 700 000 deaths per year in the United States between 2010 and 2017 [5]. IAVs are one of four genera of influenza viruses and are subdivided further into strain subtypes according to their envelope surface antigens: hemagglutinin (HA) and neuraminidase (NA) (e.g. H1N1, H3N2). IAVs also have great pandemic potential: zoonotic crossover events from swine or avian reservoirs resulted in four significant pandemics, in 1918 (H1N1), 1957 (H2N2), 1968 (H3N2), and 2009 (H1N1) [6]. In subsequent years, these strains became endemic and circulated alongside seasonal IAV strains. While mild IAV infection rapidly resolves, severe infections can result in acute respiratory distress syndrome (ARDS) and respiratory failure. Clinical lung autopsy samples display a disrupted epithelial layer, infiltration of immune cells, and elevated serum cytokine levels [7], indicating that excessive epithelial cell death and exacerbated host inflammatory responses are associated with poor clinical outcomes.

IAV readily infects and replicates in respiratory epithelial cells [8]. Cytotoxic T lymphocytes (CTLs) or Natural Killer (NK) cells kill infected epithelial cells by inducing apoptosis [9]. Alternatively, IAV-infected epithelial cells can undergo PCD, even in monoculture [10], by activating apoptotic, necroptotic or pyroptotic pathways, as recently reviewed by Laghlali et al. [8]. These PCD modalities are antiviral, as IAV-induced cell death limits viral dissemination rather than supporting new virion release [8]. The ‘guard’ hypothesis posits that multiple cell death pathways have evolved to counter pathogen-encoded antagonists that suppress specific PCD pathways [11]. For instance, when Caspase-8-dependent apoptosis is compromised, necroptosis eliminates IAV-infected cells and stimulates potent immune responses [12]. While both cell death modalities eliminate the viral replication niche, the ensuing inflammatory response is distinct.

Macrophages are sentinel innate immune cells that are a key source of inflammatory cytokines during viral infections [13]. They constantly survey the tissue environment for potential threats. Macrophages express pattern recognition receptors (PRRs) in strategic subcellular locations, which sense pathogen-associated molecular patterns (PAMPs) and damage-associated molecular patterns (DAMPs), such as are released from dying, infected cells [14]. PRR activation culminates in cytokine release. Macrophages also clear dead epithelial cells that are apically extruded from the lung epithelial barrier [15]. After ingesting apoptotic cells, macrophages activate tissue repair and regeneration gene expression programs [16]. While macrophage inflammatory responses to direct macrophage infection with IAV are well characterised [17], how macrophages integrate signals from the PAMPs and DAMPs released by neighbouring infected cells or in the ingested cells remains unclear. Macrophage cytokine responses are a crucial part of an effective immune response against IAV, yet when delayed, excessive, prolonged, or imbalanced, they can drive pathogenic inflammation and severe IAV disease [18,19]. This review will examine how macrophages sense IAV PAMPs and DAMPs released from neighbouring cells, which PCD modalities release which specific DAMPs, and how this contributes to IAV disease severity.

## Macrophage sensing of IAV

IAV can directly infect macrophages [20], which allows them to sense cell-intrinsic infection, as reviewed recently by Hulme et al. [14]. Additionally, through cell surface and endosomal PRRs, macrophages sense cell-extrinsic infection. Toll-like Receptor (TLR) 4 is a cell surface PRR best known for recognising bacterial lipopolysaccharide (LPS). LPS triggers TLR4-mediated NFκB signalling and Interferon Regulatory Factor (IRF)-dependent antiviral signalling [21]. Cell surface TLRs that sense PAMPs and DAMPs in humans include TLR1,2,5,6 and 10, while the endosomal TLRs 3,7,8,9 sense microbial nucleic acids [21]. Depending on cell-specific adaptor availability, TLR activation triggers NFκB or IRF signalling, e.g. TLR7 and TLR9 agonists trigger IFNα expression in plasmacytoid dendritic cells (DCs) but not monocytes [22]. In contrast, TLR8 activation in monocytes induces IFNα and IFNβ release [23]. TLR expression and signalling in human macrophages remain to be systematically characterised.

**Figure 1.**
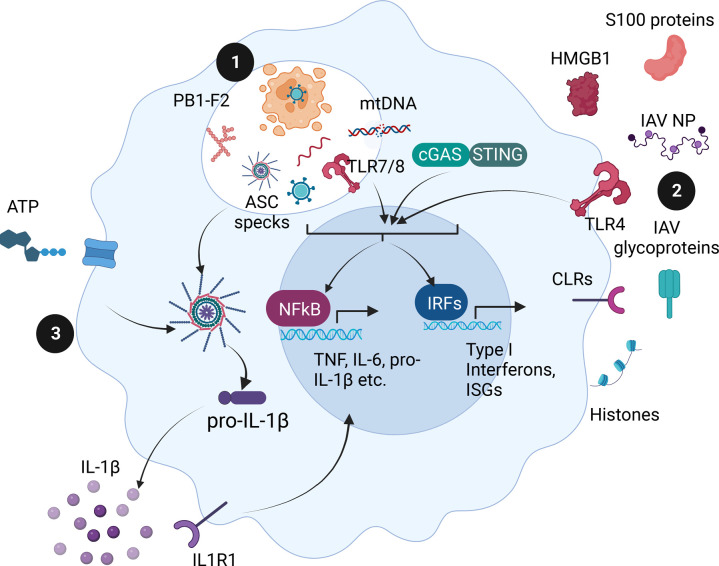
Macrophage cell-extrinsic sensing. Macrophages phagocytose dying cells and sense extracellular debris (**1**). TLR7 and TLR8 can recognise IAV ssRNA liberated from engulfed cells, while mtDNA might escape and trigger cGAS-STING signalling. Large aggregates, like PB1-F2 fibrils or ASC specks, can destabilise the lysosome and activate inflammasome signalling. Macrophage cell surface receptors sense PAMPs and DAMPs in the extracellular space (**2**). HMGB1, S100 proteins and IAV Nucleoprotein can all activate TLR4. Alternatively, CLRs recognise glycosylated IAV proteins or histones. TLR, cGAS and CLR signalling culminates in NFκB or IRF-signalling and pro-inflammatory or anti-viral gene expression. IL-1β from neighbouring cells also activates NFκB signalling. ATP or lysosomal damage activates inflammasome signalling (**3**). This figure was created using BioRender.

Dying cells release IAV proteins and nucleic acids into the extracellular space. TLR4 recognises IAV nucleoprotein and induces IL-6 and IL-1β release from mouse macrophages, though whether IAV NP triggers IFNβ release via TLR4 was not measured [24]. Meanwhile, TLR7 and TLR8 should recognise IAV genomic RNA [17], and TLR3 might recognise dsRNA intermediates present if cells die mid-replication. Additionally, various DAMPs activate TLR2 and TLR4 to trigger NFκB-dependent signalling [25]. These DAMPs include high-mobility group box 1 (HMGB1), heat-shock proteins, histones and the S100 proteins (a family of cytosolic calcium-binding proteins) [25]. Additionally, macrophage C-type Lectin Receptors, which primarily activate NFκB-dependent signalling [26], may be activated by extracellular histone proteins [27] or free viral glycoproteins [28]. As discussed below, various extracellular DAMPs, such as ATP and uric acid, activate inflammasome signalling. However, other cytosolic PRRs (RIG-I, MDA-5, ZBP-1) are unlikely to be activated during cell-extrinsic sensing unless these extracellular PAMPs and DAMPs gain access to the cytosol. Macrophages and other cells must interpret these various signals, assess the threat posed by the virus to tissue integrity, and respond accordingly. Figure 1 gives an overview of macrophage cell-extrinsic sensing during IAV infection.

## Sensing apoptotic cell death

Apoptosis is the best-known PCD pathway and is generally considered immunologically silent [4]. Apoptosis is caspase-dependent and morphologically confines any PAMPs and DAMPs within the apoptotic cell's shrinking, blebbing plasma membrane [3]. When IAV-infected cells are to be executed, CTLs or NK cells form a synapse with the target cell and release cytotoxic granules containing granzymes into the target cell cytosol. Granzyme activity triggers intrinsic apoptosis in the target cell, culminating in mitochondrial outer membrane permeabilisation, cytochrome C release and Caspase-9 activation [29]. Alternatively, CTLs and NK cells release cell death ligands such as tumour necrosis factor (TNF) and Fas ligand (FasL), which trigger extrinsic apoptosis, culminating in Caspase-8 activation [8,30]. Both granzyme-perforin and FasL pathways of CTL killing protect against IAV infection [31]. Alternatively, IAV infection alone can induce intrinsic apoptosis (reviewed in [8]). These apoptotic cells then need to be cleared from the tissue.

Consistent with an immunologically silent mode of cell death, TNF-induced apoptosis is non-lytic, with minimal release of cellular proteins into the extracellular space up to 7 h post-TNF addition [32]. Apoptotic cells are only immunologically silent if rapidly cleared from the tissue; after 15 h, apoptotic cells release equivalent cellular protein into the extracellular space as in lytic cell death. Intriguingly, apoptotic cells rapidly release histones (as early as three hours post-TNF addition) [32]. Extracellular histones can act as DAMPs to trigger pro-inflammatory signalling in macrophages [27] and promote autoantibody production [33], but whether they induce a specific apoptosis signature in macrophages is unclear.

Apoptotic cells attract macrophages by releasing soluble ‘find me’ signals, such as lysophospholipids and nucleotides, and display phosphatidylserine on their outer-facing plasma membrane as an ‘eat me’ signal [34]. Macrophages efferocytose the dying cells and degrade them in lysosomes [34]. Efferocytosis of apoptotic cells triggers an anti-inflammatory gene expression program, including expression of transforming growth factor (TGF) β and Interleukin (IL)-10, to promote tissue repair and resolution of inflammation [35]. The local tissue microenvironment largely dictates macrophage responses during efferocytosis [36], so infection will dramatically change the ensuing macrophage cytokine release and subsequent T-cell polarisation.

Alveolar macrophages can readily engulf IAV-infected epithelial cells in co-culture [37]. However, how the macrophages respond to the engulfed, IAV-infected cells and whether this is different from macrophage responses to regular apoptosis is unclear. Elegant studies comparing DC engulfment of *Citrobacter rodentium*-infected apoptotic cells with uninfected apoptotic cells demonstrated how phagocyte cytokine responses determine T-cell responses. While DCs that ingested uninfected apoptotic cells released TGFβ alone, promoting T regulatory cell induction, DCs that ingested infected apoptotic cells released both TGFβ and IL-6, resulting in Th17 cell polarisation [38]. The DCs recognised a bacterial PAMP, likely LPS, via TLRs to induce the critical IL-6 signal [38]. Whether DCs or macrophages induce a Th17 response when engulfing IAV-infected apoptotic cells should be investigated similarly. Although robust inflammatory and T-cell responses are necessary for viral clearance, excessive macrophage cytokine response and the accumulation of T-cells may drive disease. During influenza infection, T cells can also kill noninfected bystander epithelial cells, leading to irreversible lung damage [39,40]. An overactive macrophage-T cell circuit also contributes to pathology in COVID-19 [41]. A key question for the field is how to interrupt this inflammatory macrophage — T cell circuit in viral infection without compromising host defence.

Infected, dying epithelial cells also contain intact infectious virions and newly synthesised PAMPs. After ingestion, macrophage lysosomal TLRs might sense these PAMPs while degrading the apoptotic cell, such as occurs during cross-presentation in dendritic cells [17,42]. Since macrophages highly express the endo-lysosomal, single-stranded RNA (ssRNA) sensors TLR7 and TLR8 [43], and ssRNAs are key IAV-PAMPs [17], we anticipate these PRRs drive macrophage inflammatory responses upon IAV-infected epithelial cell efferocytosis. If, IAV RNAs from engulfed cells escape into the cytosol, they could trigger cytosolic PRR activation, including the RNA sensors Retinoic Inducible Gene-I (RIG-I) and Melanoma Differentiation Associated Factor -5 (MDA-5). Alternatively, efferocytosis may offer an alternate viral entry route into macrophages, and viruses may be able to enter the macrophage cytosol and start replicating. Recent studies with Severe Acute Respiratory Syndrome Coronavirus 2 (SARS-CoV-2) suggest that SARS-CoV-2 virions contained within apoptotic cells are sensed by engulfing macrophages, resulting in macrophage IL-6 and IL-1β release. In this scenario, IL-6 activation partially depended on active viral replication in the macrophages [44].

Alternatively, mitochondrial DNA (mtDNA) from dying IAV-infected cells could also drive cyclic GMP-AMP synthase (cGAS) activation, as occurs during SARS-CoV-2 infection [45]. cGAS/STING dependent sensing of SARS-CoV-2 induced cell death triggers macrophage IFNβ [45]. An intriguing cell biological question is how mtDNA from dying, engulfed cells is trafficked into the macrophage cytosol for cGAS sensing. Endo-lysosomal permeability may therefore determine macrophage PRR activation during efferocytosis and dictate the ensuing gene expression program.

## Sensing necrosis

IAV infection also reportedly triggers necrosis *in vivo* and *in vitro* [37,46,47]. Necrosis is not a PCD pathway; instead, extreme chemical or physical insults trigger terminal plasma membrane rupture [3]. Alternatively, when apoptotic cells are not promptly cleared, they undergo secondary necrosis [3]. Necrotic plasma membrane rupture releases the cellular contents into the extracellular space, including DAMPs such as HMGB1 [48]. TLR4 recognises HMGB1 and S100 proteins and induces pro-inflammatory cytokine release [25]. HMGB1 also inhibits macrophage efferocytosis [49,50], which could explain why the removal of lytic cells is less efficient than the removal of apoptotic cells [51,52]. TLR4 inhibition is protective in mouse models of IAV, highlighting the potential role of DAMPs in IAV-induced inflammation [53]. Similarly, impaired dead cell clearance and the accumulation of necrotic cells are linked to various chronic autoinflammatory diseases and exacerbated lung pathology during IAV infection [15,54]. Thus, the timing and efficiency of the dead cell clean-up also dictate the immune response to dying cells.

## Sensing necroptosis

Necroptosis is another PCD pathway activated during IAV infection [55–58]. Critically, necroptosis is caspase-independent. Instead, necroptosis activates MLKL (Mixed Lineage Kinase domain-like pseudokinase), such that it oligomerises into pores in the plasma membrane, rapidly releasing cellular contents [59,60]. Several recent studies have mapped the molecular pathways by which IAV activates necroptosis. Z-DNA binding protein 1 (ZBP1) recognises Z-RNAs produced during IAV replication and activates receptor-induced kinase 3 (RIPK3). RIPK3 can then activate Caspase-8-dependent apoptosis, or, if this is inhibited (e.g. by pan-caspase inhibitors or by viral antagonism), RIPK3 phosphorylates MLKL to trigger necroptosis [55–58,61]. Studies by Shubina et al. suggest an IAV-infected cell will commit to either Caspase-8-dependent apoptosis or MLKL-dependent-necroptosis [12], though how the cell makes that decision is less clear. Whether ZBP1 signalling is protective or pathogenic likely depends on the inoculum dose. At low IAV doses, ZBP1 knockout mice are more susceptible to infection, while at high doses, knockout mice are equally susceptible as WT mice [58]. Given that ZBP1 activation also drives IFN and pro-inflammatory cytokine release [57], its role as protective or pathogenic is challenging to determine. Similarly, the physiological role of necroptosis and MLKL during IAV infection remains controversial, with one study reporting no role for MLKL [62] and others suggesting it contributes to disease pathology [58]. These discrepancies may reflect confounding effects from the IAV strain or mouse genetic backgrounds.

IAV replicates in the nucleus yet is sensed by cytosolic RNA sensors, such as RIG-I and ZBP1. A pool of nuclear RIG-I [63], or translocated ZBP1 [58], recognises nuclear IAV replication products or Z-RNAs. How RIG-I and ZBP1 recruit their cytosolic adaptors into the nucleus for continued signalling is less clear. If ZBP1 activates necroptosis, MLKL is recruited into the nucleus to begin ‘inside-out' signalling, rupturing the nucleus before it ruptures the plasma membrane. Zhang et al. [58] found that this MLKL-mediated nuclear rupture released nuclear-specific DAMPs, such as HMGB1 and DNA, driving neutrophil recruitment and neutrophil activation in IAV infection *in vivo* [58]. In contrast, IAV-induced ZBP1-Caspase-8 dependent cell death does not trigger the same nuclear herniation nor pathology *in vivo* [58], suggesting that only the necroptotic signature is pro-inflammatory. Which cells sense these necroptotic DAMPs and how has not yet been elucidated.

So far, it has been difficult to dissect the net effect of reduced pro-inflammatory cytokines versus increased necroptotic DAMP release on macrophage and tissue responses, especially during infection. A systematic comparison of TNF-induced apoptosis with necroptosis showed that necroptosis uniquely limits conventional pro-inflammatory cytokine secretion while releasing HMGB1 and lysosomal proteins [32]. Whether IAV-induced necroptosis similarly limits conventional cytokine secretion and whether necroptotic signalling is pro-inflammatory or anti-inflammatory *in vivo* is unclear*.* Supernatants from live, TNF-stimulated, cytokine-producing cells promoted inflammation in mice, while supernatants from necroptotic TNF-stimulated cells did not [64]. In contrast, macrophage efferocytosis of necroptotic cells triggered robust IL-6 and TNF induction compared with efferocytosis of apoptotic cells in *in vitro* assays [65]. Whether specifically inhibiting necroptosis reduces pathogenic inflammation during IAV infection merits further investigation.

## Sensing pyroptosis

Pyroptosis is a highly pro-inflammatory form of cell death activated downstream of inflammasome signalling. Inflammasomes are cytosolic signalling platforms that process the IL-1β, IL-18 and the pore-forming protein gasdermin D (GSDMD) into their mature, active forms. Briefly, inflammasome signalling is a two-step process. During the first step, PRRs recognise PAMPs or DAMPs and activate NFκB signalling, increasing pro-IL-1β expression and priming the inflammasome sensors for activation [66]. A second signal, such as an IAV infection, triggers oligomerisation of the inflammasome sensor scaffold. NLR family pyrin domain containing 3 (NLRP3) is a key sensor during IAV infection [67,68]. The adaptor apoptosis-associated speck-like protein containing a CARD (ASC) is recruited to the inflammasome sensor and then proceeds to oligomerise into filaments that coalesce into an ASC ‘speck' [69]. ASC recruits Caspase-1, which homo-dimerises and self-cleaves into its active form [70], where it can then process IL-1β, IL-18 and GSDMD. GSDMD forms pores in the plasma membrane, and low molecular weight cellular proteins, including mature IL-1β and IL-18, and HMGB1 and Galectin 3 (LGAL3) exit through these pores [71]. GSDMD pore formation eventually promotes terminal cell rupture, which releases large cellular proteins, such as the 147 kDa lactate dehydrogenase (LDH) complex [71], or ASC specks themselves [72], into the extracellular space.

Mature IL-1β and IL-18 are incredibly potent pro-inflammatory cytokines [73]. IL-1β signals through its receptor IL1R1, which is widely expressed in various cell types and promotes the induction of NFκB-dependent cytokines [73]. IL-18 promotes IFN-γ production from T cells and NK cells [74]. While some cytokines (e.g. IL-1β and TNF) and DAMPs (e.g. HMGB1) prime the inflammasome, many of the DAMPs released by pyroptosis, such as ATP and uric acid, can themselves activate the NLRP3 inflammasome. Extracellular ASC specks can also seed new inflammasome activation in neighbouring cells [72]. While this amplifying loop is critical for inducing protective adaptive immune responses, it can promote pathogenic inflammation. Accordingly, aberrant inflammasome activation is implicated in the pathogenesis of many diseases, including multiple sclerosis, atherosclerosis, Alzheimer's, and Parkinson's disease [75].

NLRP3 and inflammasome signalling is protective early during IAV infection [76,77] and initiates effective adaptive immunity [78]. Elegant studies with an NLRP3 inhibitor showed that NLRP3 signalling drives disease later in infection [79], likely by amplifying pathogenic inflammation. Some IAV strains can directly infect murine macrophages, activating NLRP3 [8] in a cell-intrinsic manner. NLRP3 may sense either incoming or replicating IAV RNA or viral ribonucleoprotein complexes (vRNPs) via ZBP1 [57,76]. NLRP3 also senses pH changes in the Golgi compartment caused by the IAV M2 protein during new viral protein synthesis and virion assembly [67]. Alternatively, when macrophages phagocytose IAV PB1-F2 protein aggregates (released from dying cells), these aggregates destabilise the macrophage lysosomes and activate NLRP3 [68].

Inflammasome activation in IAV-infected epithelial cells is less well characterised. The restriction factor MxA reportedly nucleates ASC and enables IL-1β release from airway epithelial cells upon H1N1 infection, though cell death was not assessed [80]. In contrast, infection of airway epithelial cells with the highly pathogenic IAV strain H7N9 triggered Gasdermin E (GSDME)-dependent pyroptosis [81], though the upstream mechanism is unclear. In mice, GSDME deficiency protected against H7N9-induced lethal cytokine storm [81], indicating that the DAMPs released during pyroptosis amplify dangerous inflammation.

**Figure 2.**
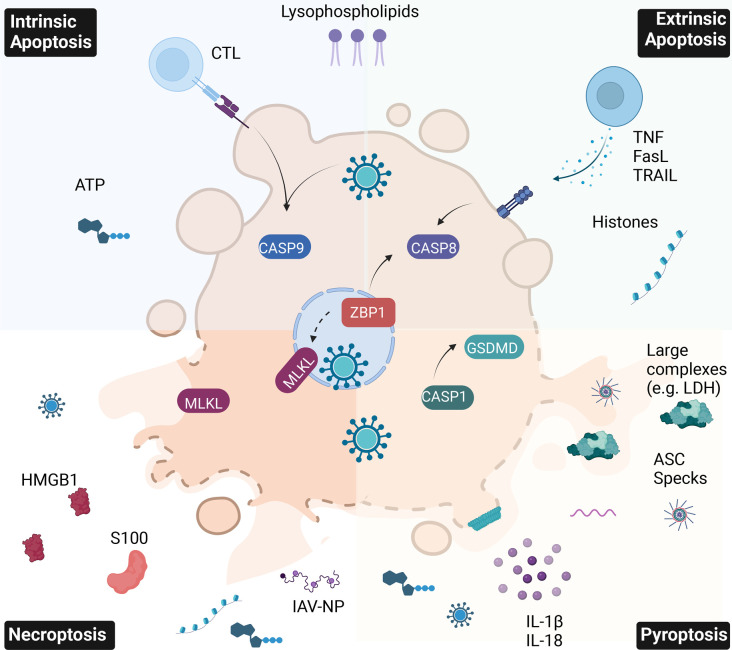
Specific PAMP and DAMP release from major PCD pathways during IAV infection. This figure gives a simplified overview of PCD pathways. Clockwise from upper left: Intrinsic Apoptosis, triggered by direct IAV infection or CTL or NK-cell granule exocytosis, culminates in Caspase-9 activation. Extrinsic Apoptosis, triggered by cell death ligands (TNF, FasL and TRAIL), culminates in Caspase-8 activation. These proceed to activate the executioner Caspases 3 and 7. Cellular contents are mostly sequestered in apoptotic bodies with intact plasma membranes, though histones, ATP, and phospholipids are released as ‘find me’ signals. Pyroptosis, triggered upon inflammasome-dependent Caspase-1 activation, culminates in GSDMD pore formation in the plasma membrane. Low molecular weight PAMPs, DAMPs and cytokines (especially IL-1β and IL-18) exit through GSDMD pores, while terminal cell membrane rupture releases high molecular weight protein complexes like LDH and ASC specks. Necroptosis, triggered by ZBP1 recognition of IAV Z-RNA, culminates in MLKL pores rupturing the plasma membrane. This releases cellular DAMPs such as HMGB1, S100 proteins and ATP, and IAV PAMPs, like IAV nucleoproteins and ssRNAs. This figure was created using BioRender.

Macrophages also activate the inflammasome in response to cell-extrinsic infection. IAV PB1-F2 and M2 also trigger oxidised DNA release from macrophages into the extracellular space. The absent from melanoma 2 (AIM2) inflammasome in neighbouring cells senses this oxidised DNA, triggering macrophage IL-1β release [82]. A similar phenomenon was recently observed in SARS-CoV-2 infection, where extracellular DNA released from dying, infected epithelial cells triggers macrophage inflammasome activation [83]. Cell extrinsic sensing of IAV infection to drive macrophage pyroptosis may therefore play a critical role in infections with IAV strains that do not infect macrophages. Figure 2 gives an overview of the distinct DAMPs released by each PCD pathway.

Some key questions must be answered to successfully target inflammasome signalling during IAV infection without compromising host defence. Does cell-intrinsic inflammasome activation induce a distinct inflammatory signature compared with cell-extrinsic activation? What role does GSDMD-dependent pyroptosis play in IAV infection? Does epithelial cell inflammasome activation play a different role in infection control than macrophage inflammasome activation? Pyroptotic macrophages appear to release DAMPs before IL-1β and IL-18 release [71]. Presumably, small viral PAMPs will exit the cell through GSDMD pores, while cell rupture will release larger viral PAMPs. How do neighbouring cells integrate these distinct temporal signals and relative DAMP and cytokine concentrations to shape the ensuing inflammatory response? Answering these questions will accelerate the use of inflammasome-targeting therapies as immunomodulators in viral infections.

## Conclusions and future directions

One of the most exciting new aspects of PCD biology is how this process is regulated to control DAMP release. Recent studies show that the cell membrane protein NINJ1 mediates plasma membrane rupture, which is the final stage of lytic cell death for both pyroptosis and necroptosis [84]. NINJ1 deficiency increases susceptibility to bacterial infection, presumably by limiting critical inflammatory responses initiated by DAMPs. NINJ1 activity competes with active membrane repair, mediated by the endosomal sorting complexes required for transport (ESCRT) proteins. ESCRT-III proteins can repair MLKL or GSDMD-induced pores in cellular membranes, preventing terminal plasma membrane rupture [85,86]. For necroptosis, this prolongs cytokine release and potentiates adaptive immune responses through CD8 cross-priming, while for pyroptosis, this impairs IL-1β and IL-18 release and limits inflammation [85,86]. How infected epithelial cells regulate NINJ1 and ESCRT III activity and whether targeting these pathways could fine-tune downstream inflammatory responses are exciting questions for future research.

Does it matter how a cell dies so long as it is eliminated? The guard hypothesis suggests that we have evolved these distinct PCD modalities to counteract pathogen-encoded antagonists of PCD [11]. Detailed comparisons of apoptotic, necroptotic and pyroptotic cells show that these cell death modalities release distinctive patterns of DAMPs and cytokines with specific timing and magnitude. We speculate that in addition to ensuring a cell dies, lytic cell death modalities, such as necroptosis and pyroptosis, also communicate the relative virulence of an infecting pathogen and the threat it poses to neighbouring cells. This is analogous to the PRR threat assessment paradigm, where PRR location determines the strength of the ensuing inflammatory response [87,88]. Thus, neighbouring macrophages, among other cells, might sense these DAMPs and PAMPs, associated with specific cell death modalities and escalate the immune response accordingly. As different PAMPs and DAMPs engage PRRs with distinct signalling outcomes, the extracellular ‘soup’ composition will dictate which signalling pathways are activated, e.g. NFκB-dependent, antiviral or inflammasome-dependent pathways.

The inhibition or modulation of specific cell death signalling pathways is a potential strategy to alleviate IAV-induced immunopathology. However, such an approach must carefully consider whether inhibiting cell death enhances IAV replication and disrupts host tissue homeostasis. This strategy will also heavily depend on the infecting IAV strain, as different IAV strains induce different cell death modalities in different cell types. Viral tropism, viral antagonism of cell death pathways, or virulence factor expression may dictate which PCD pathway should be targeted. For example, seasonal H1N1 strains induce DC necroptotic cell death, while pandemic H1N1 strains suppress necroptosis [89], and PB1-F2 from pathogenic IAV but not seasonal IAV triggers pyroptotic responses [68]. The antagonism of signalling pathways involved in DAMP/cytokine recognition could be a more feasible approach to prevent lung hyperinflammation during the disseminated stage of IAV infection without compromising host defence mechanisms. Future exciting research delineating the relationship between infection, cell death, inflammation, and disease prognosis will facilitate the development of novel therapeutic approaches for viral diseases.

## Perspectives

- Targeting specific cell death pathways may limit virus-induced inflammation without compromising host defence, which may yield new drug targets for treating viral infections
- Apoptosis, necroptosis, and pyroptosis each release distinct host cell molecules, which neighbouring cells can sense as a sign of damage and danger, in addition to microbial components to tailor the immune response.
- While we deeply understand the molecular pathways of ‘how’ a cell dies during infection, future research will determine the consequences of each cell death modality on neighbouring cells.

## Open Access

Open access for this article was enabled by the participation of University of Queensland in an all-inclusive *Read & Publish* agreement with Portland Press and the Biochemical Society under a transformative agreement with CAUL.

## Abbreviations

ARDS: acute respiratory distress syndrome
cGAS: cyclic GMP-AMP synthase
CTL: cytotoxic T lymphocyte
DAMPs: damage-associated molecular patterns
FasL: Fas ligand
GSDMD: Gasdermin D
GSDME: Gasdermin E
HA: hemagglutinin
HMGB1: high mobility group box 1
IAV: influenza A virus
IL: Interleukin
IRF: Interferon Regulatory Factor
LDH: lactate dehydrogenase
LGAL3: Galectin 3
LPS: lipopolysaccharide
MDA-5: Melanoma Differentiation Associated Factor-5
MLKL: Mixed Lineage Kinase domain-like pseudokinase
mtDNA: mitochondrial DNA
NA: neuraminidase
NF-κB: nuclear factor kappa B
NK: Natural Killer
NLRP3: NLR family pyrin domain containing 3
PAMPs: pathogen-associated molecular patterns
PCD: programmed cell death
PRR: pattern recognition receptor
RIG-I: Retinoic Inducible Gene-I
RIPK3: receptor-induced kinase 3
SARS-CoV-2: Severe Acute Respiratory Syndrome Coronavirus 2
ssRNA: single-stranded RNA
TGF: transforming growth factor
TLR: toll-like receptor
TNF: tumour necrosis factor
vRNPs: viral ribonucleoprotein complexes
WT: wild-type
ZBP1: Z-DNA binding protein 1
